# Supplementary material for: Unraveling Mechanisms and Impact of Microbial Recruitment on Oilseed Rape (Brassica napus L.) and the Rhizosphere Mediated by Plant Growth-Promoting Rhizobacteria
Source: Microorganisms. 2021 Jan 12;9(1):161. doi: 10.3390/microorganisms9010161 (PMC7828142; doi:10.3390/microorganisms9010161)
Supplement: Supplementary file 1 [file microorganisms-09-00161-s001.pdf]

SUPPLEMENTARY INFORMATION

Unravelling mechanisms and impact of microbial recruitment on Oilseed rape (*Brassica napus L.*) and the rhizosphere mediated by plant growth-promoting rhizobacteria

Ying Liu[1,2], Jie Gao[2,3], Zhihui Bai[2,3], Shanghua Wu[2,3], Xianglong Li[2,3], Na Wang[2,3], Xiongfeng Du[2,3], Haonan Fan[2,3], Guoqiang Zhuang[2,3], Tsing Bohu[4] and Xuliang Zhuang[2,3,*]

[1] School of Life Sciences, University of Science and Technology of China, Hefei 230026, China;
[2] CAS Key Laboratory of Environmental Biotechnology, Research Center for Eco-Environmental Sciences, Chinese Academy of Sciences, Beijing 100085, China;
[3] College of Resources and Environment, University of Chinese Academy of Sciences, Beijing 100049, China;
[4] CSIRO Mineral Resources, Kensington, Western Australia 6151, Australia.
* Correspondence: xlzhuang@rcees.ac.cn; Tel.: +86-10-62849193

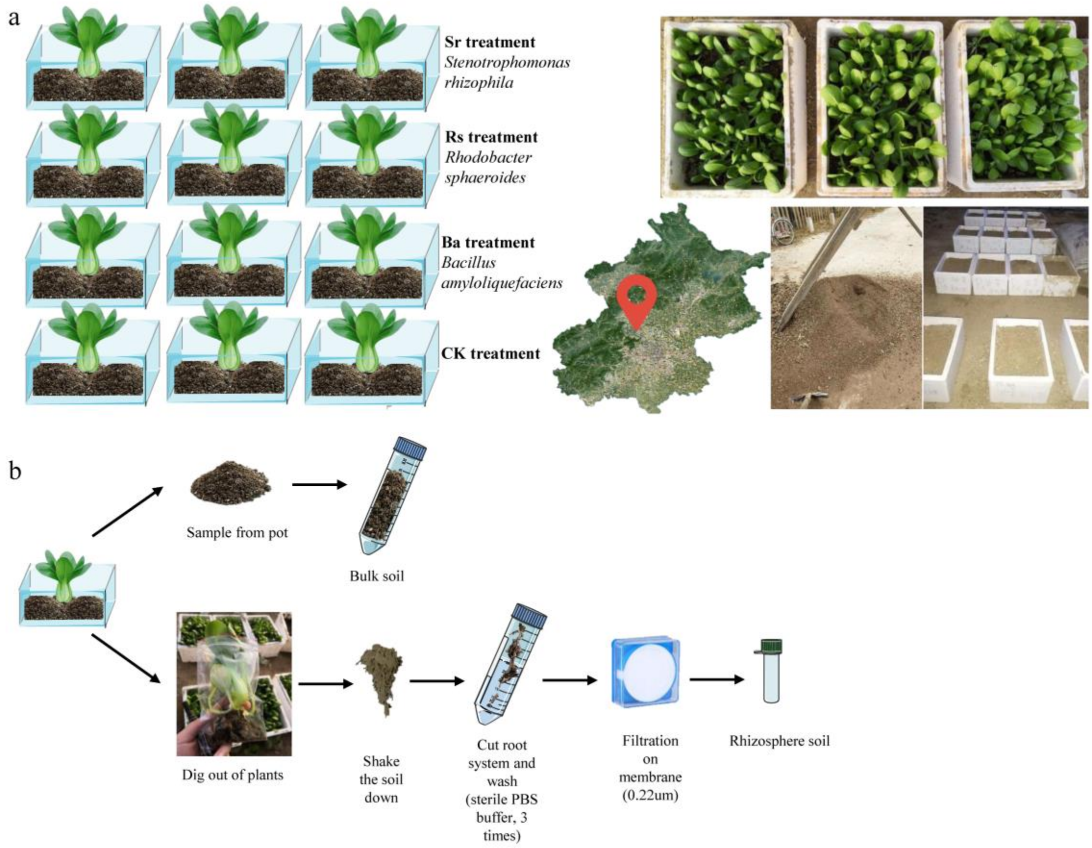

Fig. S1. (a) Diagram and photographs (before planting and plant growth final stage) of PGPR treatments in greenhouse; (b) Fractionation protocol. For each biological replicate (n = 3), five individual plants were dug out from the pots and samples were fractionated into bulk soil and rhizosphere soil.

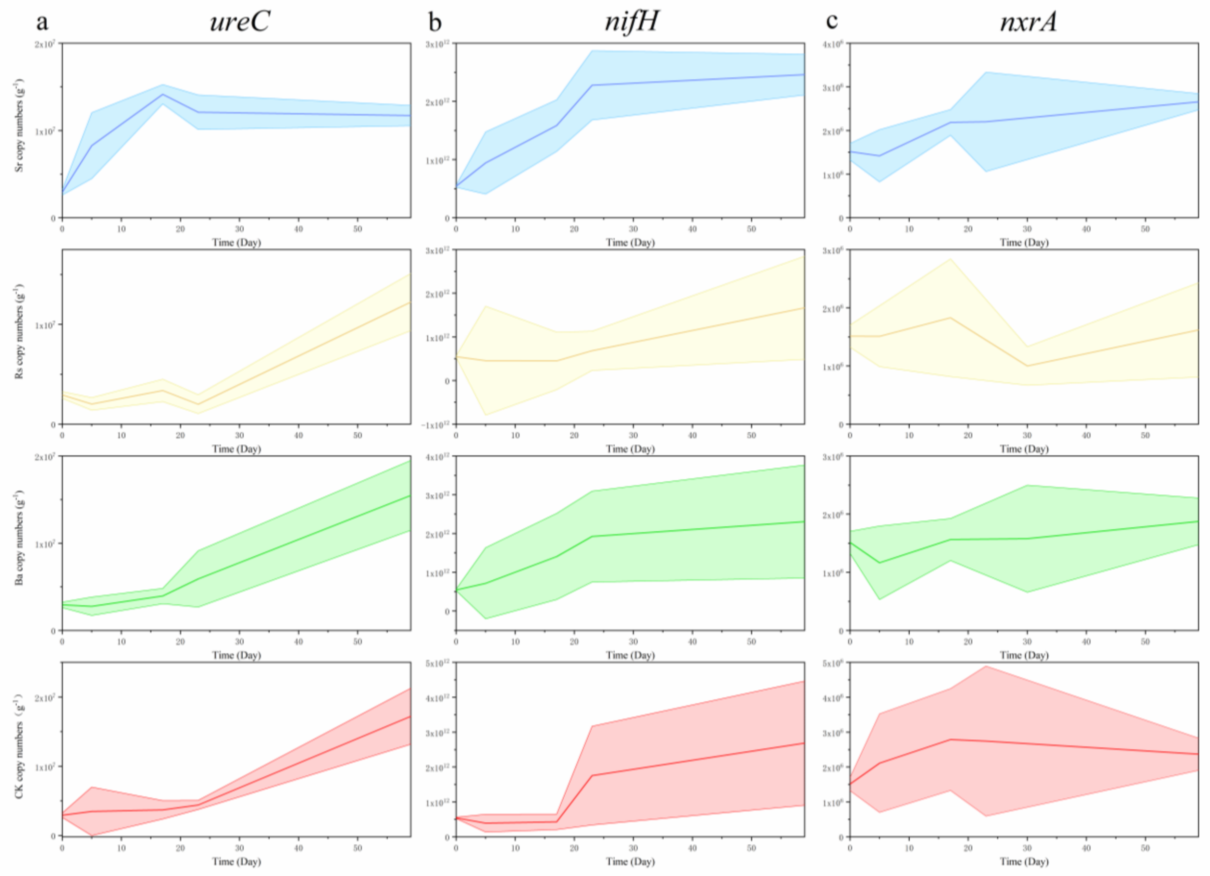

Fig. S2. Quantitative curve trends from day 5 to day 59 of *ureC* (a), *nifH* (b), and *nxrA* (c). The peripheral curves in each graph represent the error (SD).

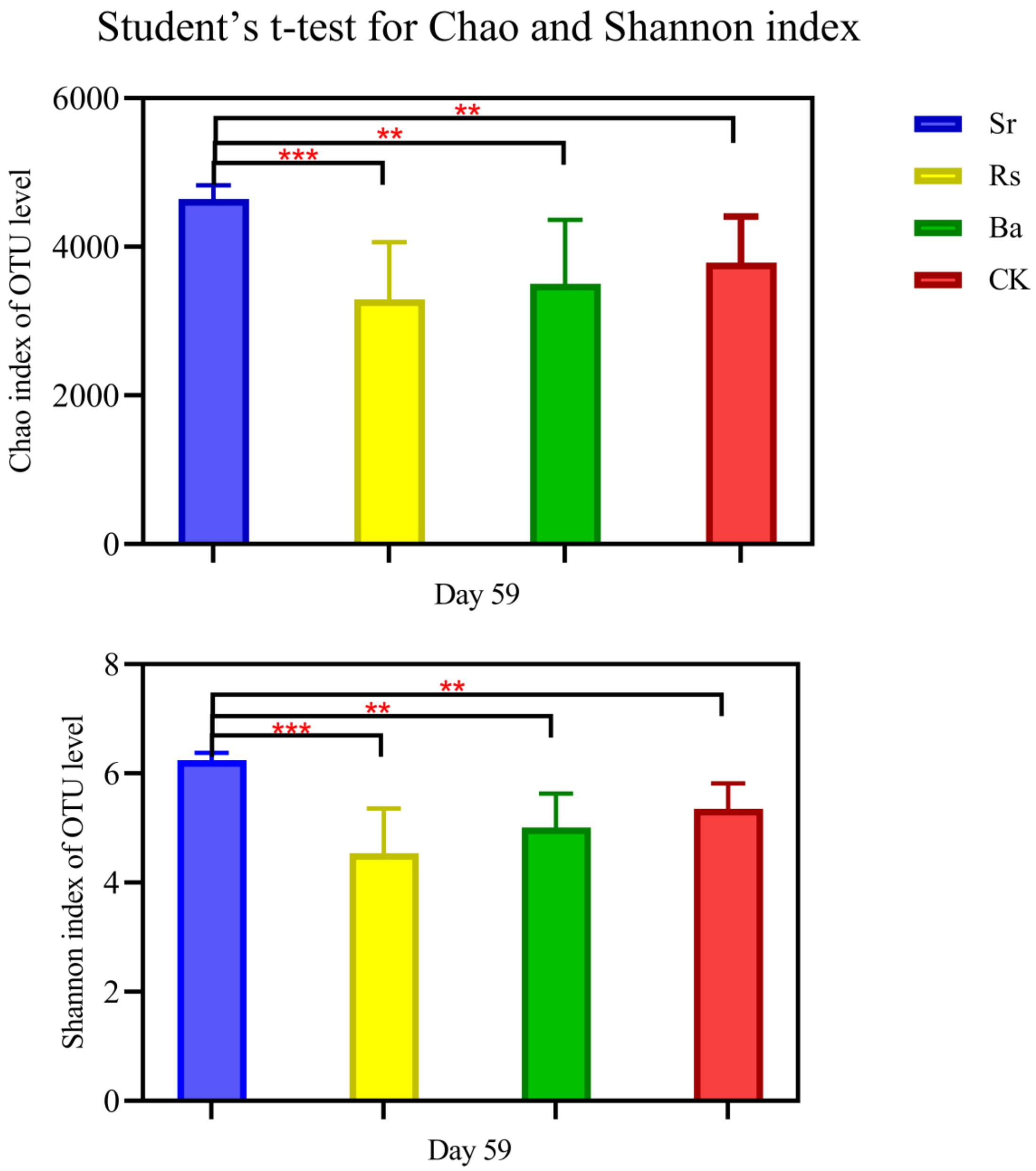

Fig. S3. Alpha-diversity of the four treatments on Day 59. Student's t-test, p-values (*P<0.05; **P<0.01, ***P<0.001).

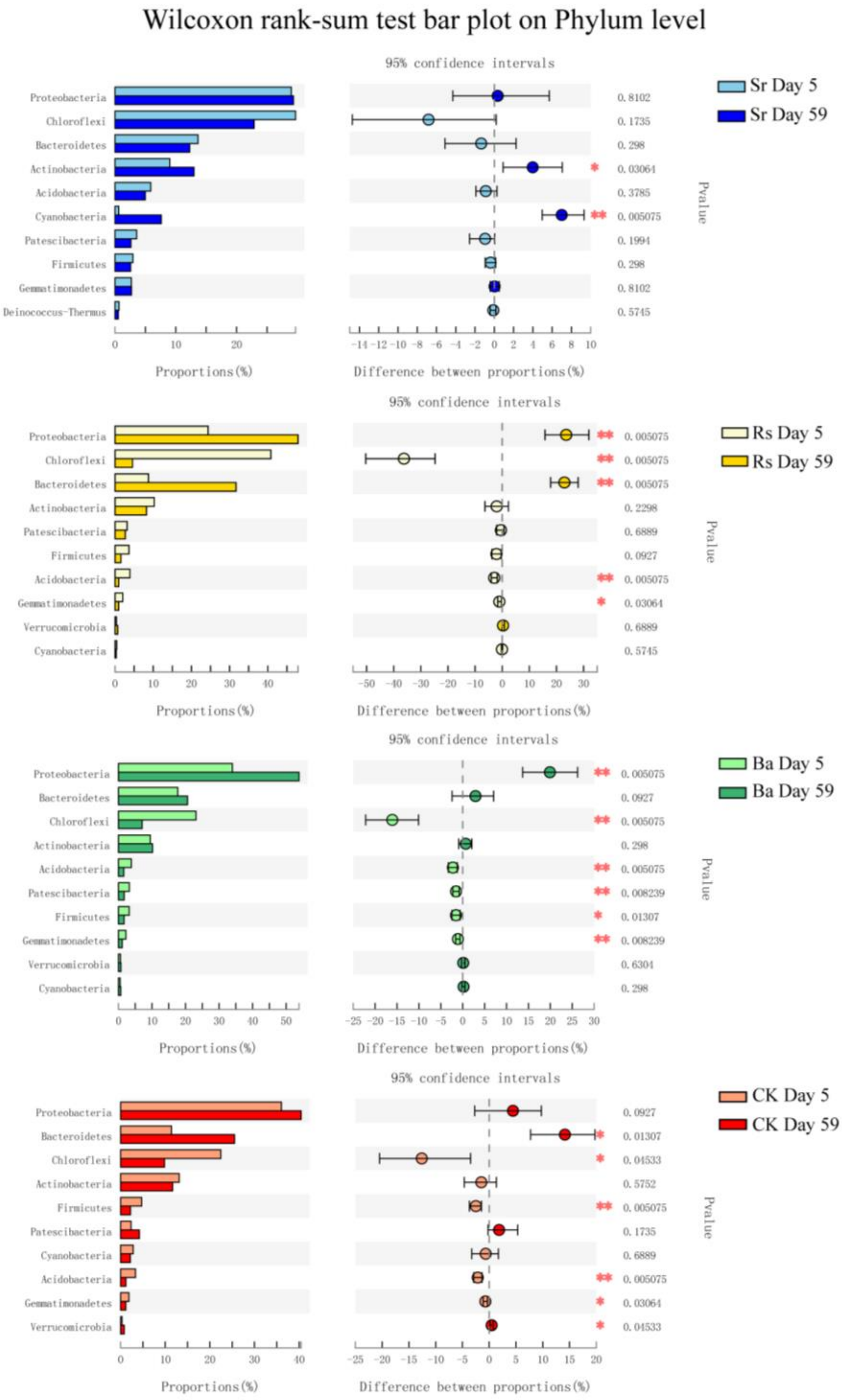

Fig. S4. Differences in the relative abundance of the top ten rhizobacterial phyla among treatments over the course of the experiment as based on Wilcoxon rank-sum test. Extended error bar plots denote statistically significant features along with the p-values (*P<0.05; **P<0.01, ***P<0.001), effect sizes, and confidence intervals (95%).

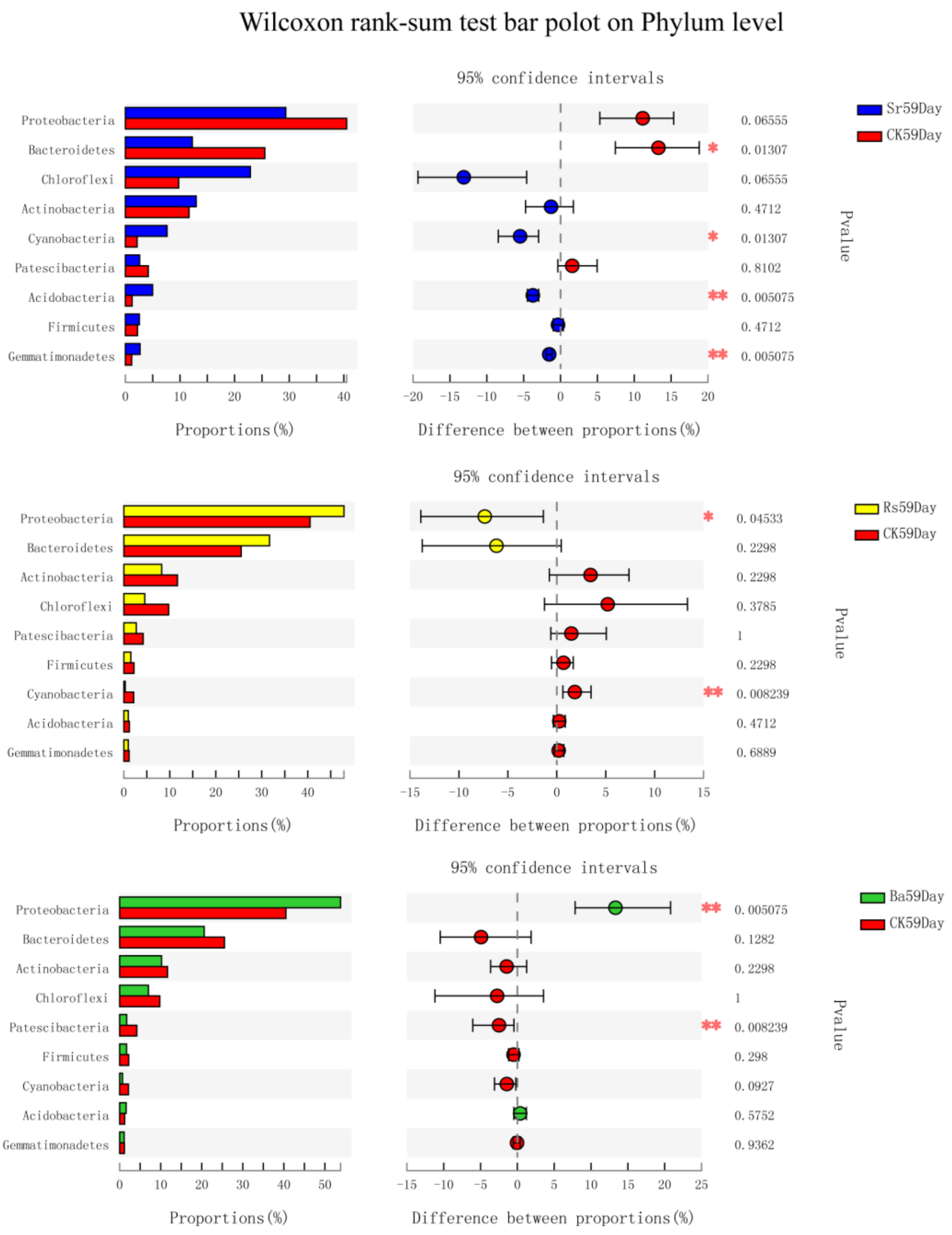

Fig. S5. Differences in the relative abundance of the top nine rhizobacterial phyla among treatments on day 95 of growth experiment as based on Wilcoxon rank-sum test. Extended error bar plots denote statistically significant features along with the p-values (*P<0.05; **P<0.01, ***P<0.001), effect sizes, and confidence intervals (95%).

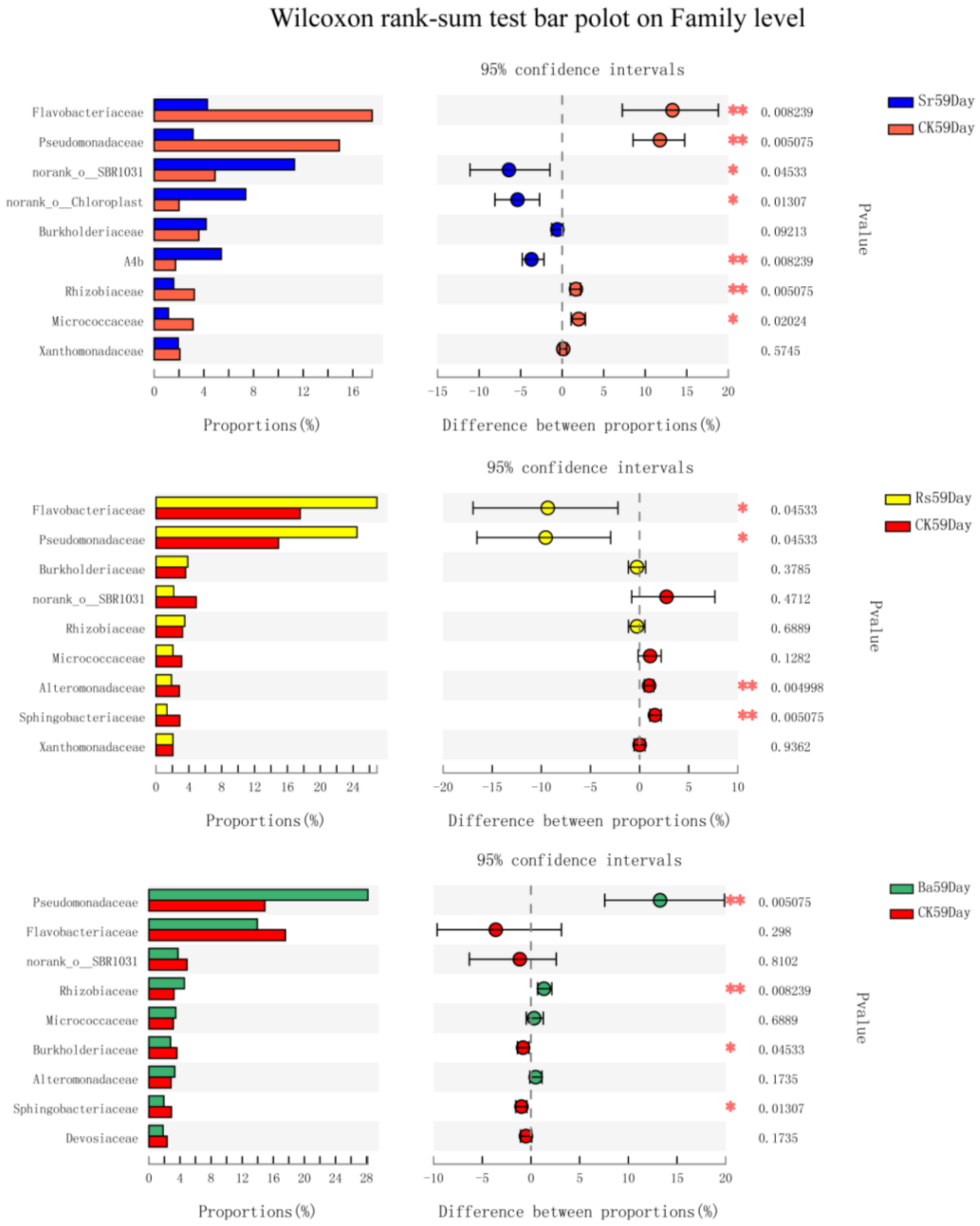

Fig. S6. Differences in the relative abundance of the top nine rhizobacterial families among treatments on day 95 of growth experiment as based on Wilcoxon rank-sum test. Extended error bar plots denote statistically significant features along with the p-values (*P<0.05; **P<0.01, ***P<0.001), effect sizes, and confidence intervals (95%).

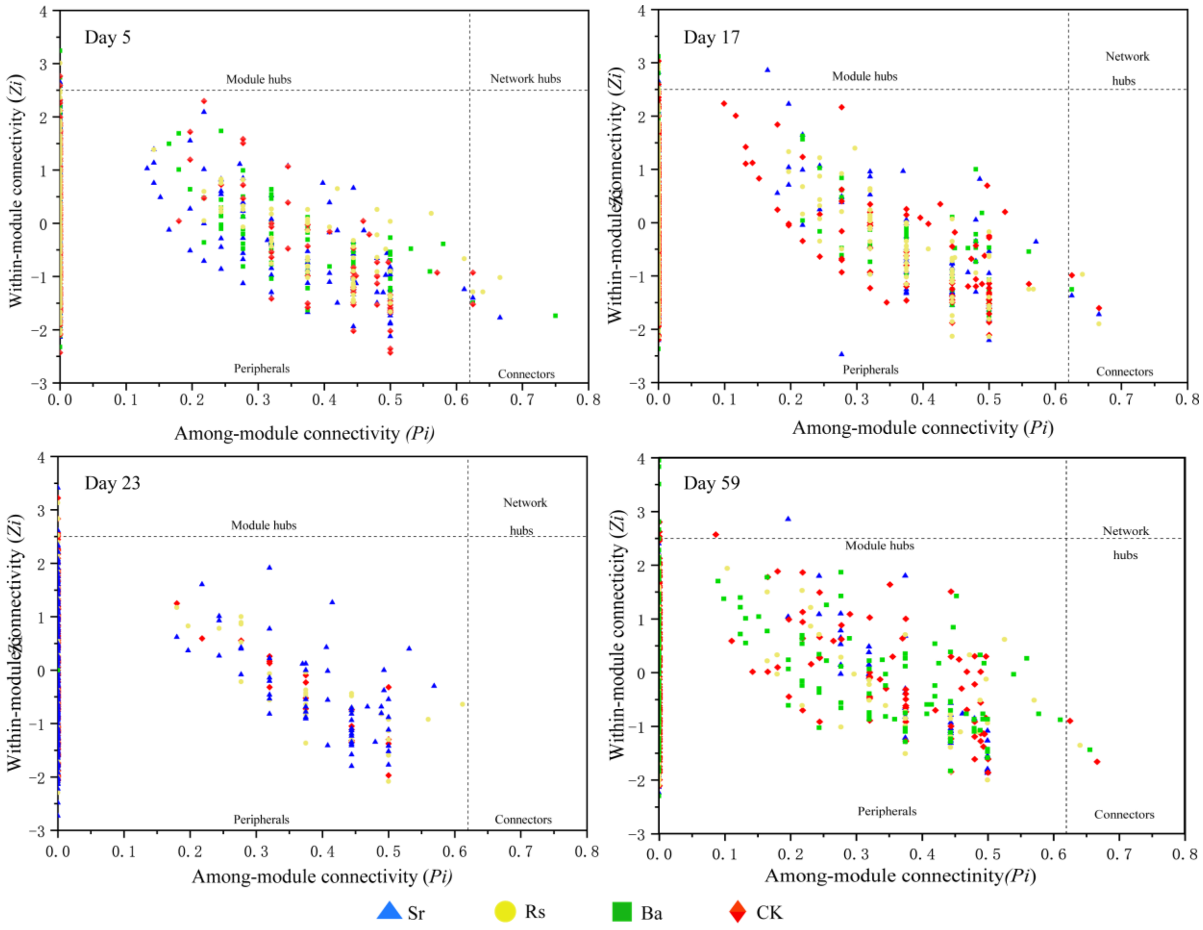

Fig. S7. Distribution of Zi and Pi values of all nodes with four treatments during the four plant growth stages.

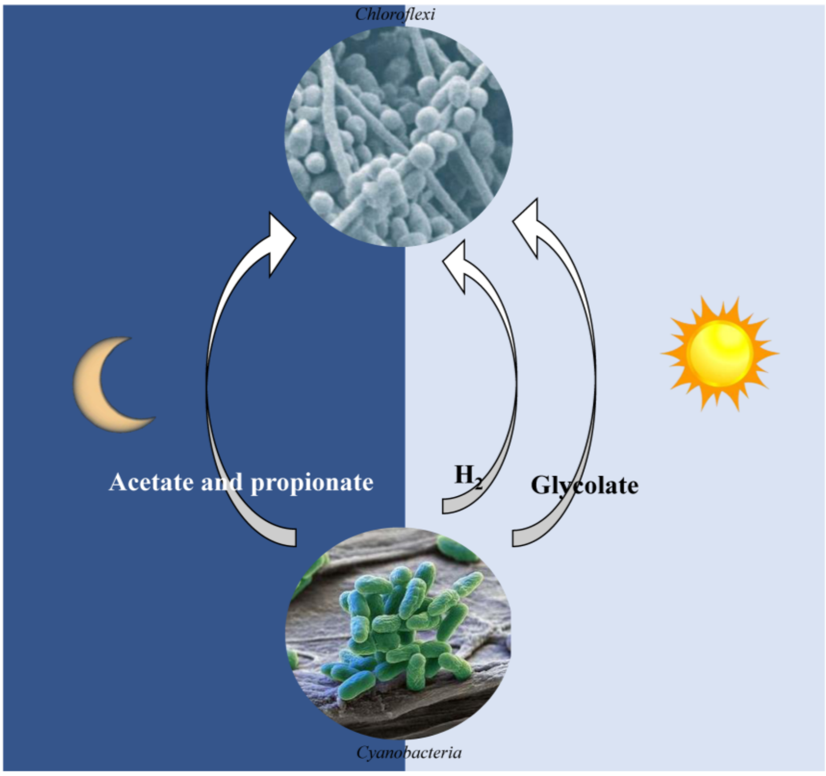

Fig. S8. Diagram of mutualism relationship between *Chloroflexi* and *Cyanobacteria*. (The image of *Chloroflexi* is from Lab Microbial Systems Ecology, Department of Microbiology, TUM, Germany; the image of *Cyanobacteria* is from Eye of Science/Science Photo Library.)

Table S1. Beneficial characteristics of the PGPR.

Promoting ability	Detection method	*B.amyloliquefaciens*	*S. rhizophila*	*R. sphaeroides*
Nitrogen fixation	Burks Medium	+	-	-
Phosphorus solubilization	Pikovskaya's Broth medium	+	+	-
Siderophore production	Chrome azurol S (CAS) agar plate	-	+	+
Indoleacetic acid (IAA) detection	Salkowski assay	-	-	-
ACC deaminase activity	Spectrophotometry colorimetry	+	-	-
Biofilm synthesis	Crystal violet staining	+	+	-

- means inability, + means ability.

Table S2. Topological properties of pMENs and random networks obtained among the rhizosphere of the four treatments during four plant growth stages, and their ZP-plot quantities.

Empirical network									Random network			ZP-plot	
Treatment	Time	Network Indexes	Total nodes	Total links	Average degree (avgK)	Average clustering coefficient (avgCC)	Average path distance (GD)	Modularity	Average path (GD)	Average clustering coefficient (avgCC)	Modularity (M)	module hubs	Connectors
Sr	5 Day	0.98	1274	3004	4.716	0.493	18.168	0.896	4.511 ± 0.019	0.007 ± 0.002	0.461 ± 0.003	3	2
Rs	5 Day	0.98	934	1723	3.69	0.425	17.948	0.912	5.102 ± 0.035	0.005 ± 0.001	0.552 ± 0.004	1	3
Ba	5 Day	0.96	1148	2498	4.352	0.523	17.678	0.909	4.742 ± 0.022	0.006 ± 0.002	0.490 ± 0.004	3	2
CK	5 Day	0.98	1190	2429	4.082	0.486	19.556	0.92	4.940 ± 0.026	0.005 ± 0.002	0.514 ± 0.004	3	2
Sr	17 Day	0.98	1144	2038	3.563	0.424	17.233	0.915	5.413 ± 0.030	0.004 ± 0.001	0.571 ± 0.004	3	2
Rs	17 Day	0.98	1300	2561	3.94	0.468	18.782	0.929	5.189 ± 0.023	0.004 ± 0.001	0.529 ± 0.004	4	2
Ba	17 Day	0.96	1151	2008	3.489	0.476	21.517	0.918	5.440 ± 0.035	0.005 ± 0.002	0.579 ± 0.004	6	1
CK	17 Day	0.98	1187	2607	4.393	0.479	13.977	0.898	4.655 ± 0.022	0.006 ± 0.002	0.486 ± 0.003	5	2
Sr	23 Day	0.98	1130	2279	4.034	0.456	16.016	0.901	4.912 ± 0.028	0.005 ± 0.001	0.517 ± 0.004	6	0
Rs	23 Day	0.98	1090	1765	3.239	0.451	21.71	0.953	5.859 ± 0.034	0.004 ± 0.001	0.615 ± 0.003	3	0
Ba	23 Day	0.99	448	325	1.451	0.299	1	0.989	6.715 ± 1.582	0.003 ± 0.002	0.958 ± 0.005	0	0
CK	23 Day	0.98	1004	1505	2.998	0.44	12.261	0.961	6.181 ± 0.044	0.004 ± 0.002	0.651 ± 0.004	9	0
Sr	59 Day	0.98	1134	1824	3.217	0.464	19.497	0.955	6.014±0.036	0.003±0.002	0.619±0.004	7	0
Rs	59 Day	0.96	489	1278	5.227	0.505	12.004	0.821	3.797±0.031	0.018±0.003	0.416±0.004	0	1
Ba	59 Day	0.95	601	1853	6.166	0.532	8.854	0.801	3.641± 0.024	0.020±0.003	0.374±0.004	6	3
CK	59 Day	0.96	714	2122	5.944	0.542	10.127	0.786	3.729±0.023	0.019± 0.003	0.382±0.004	5	2
